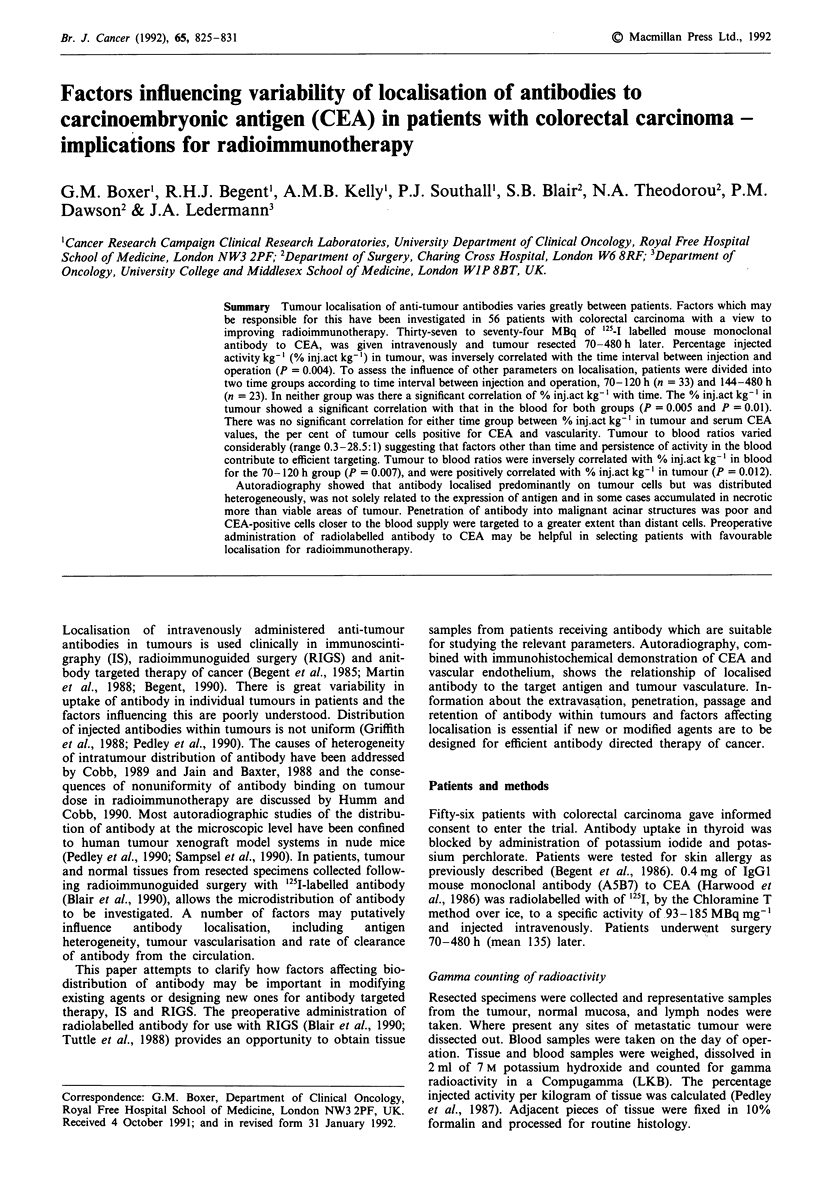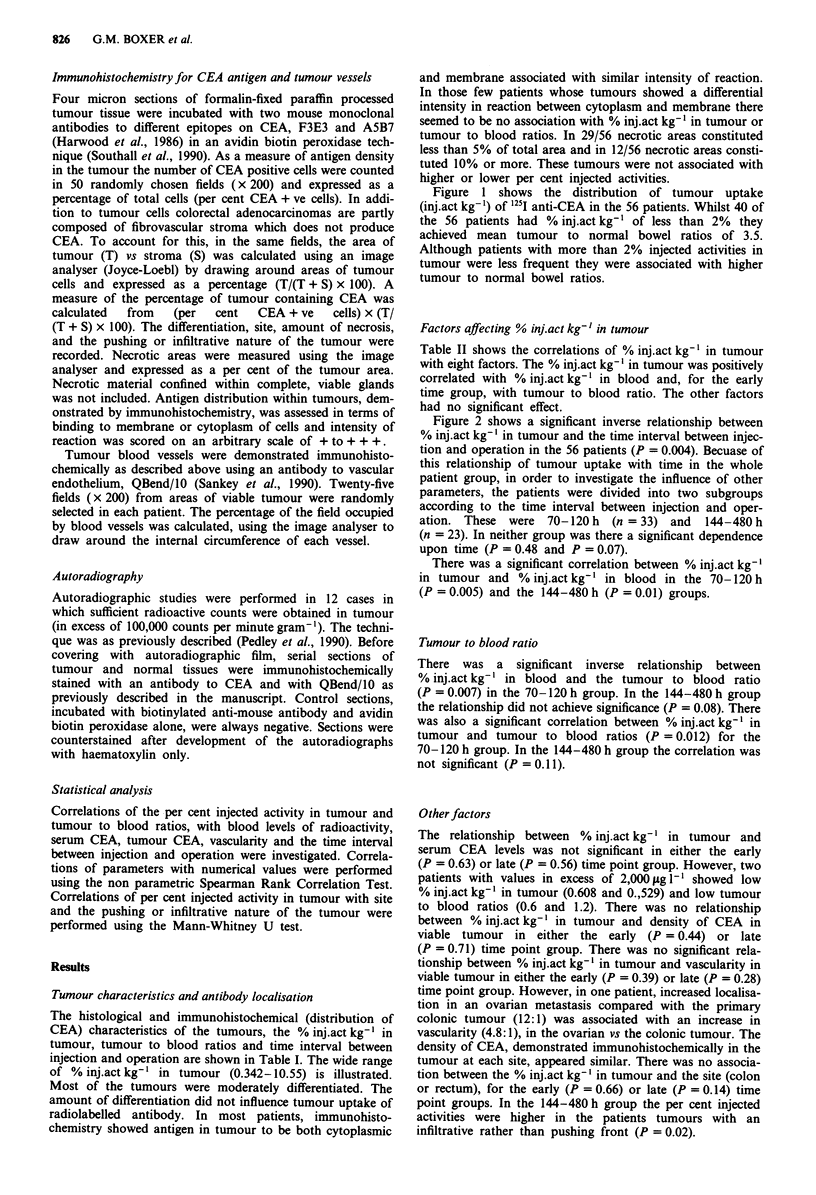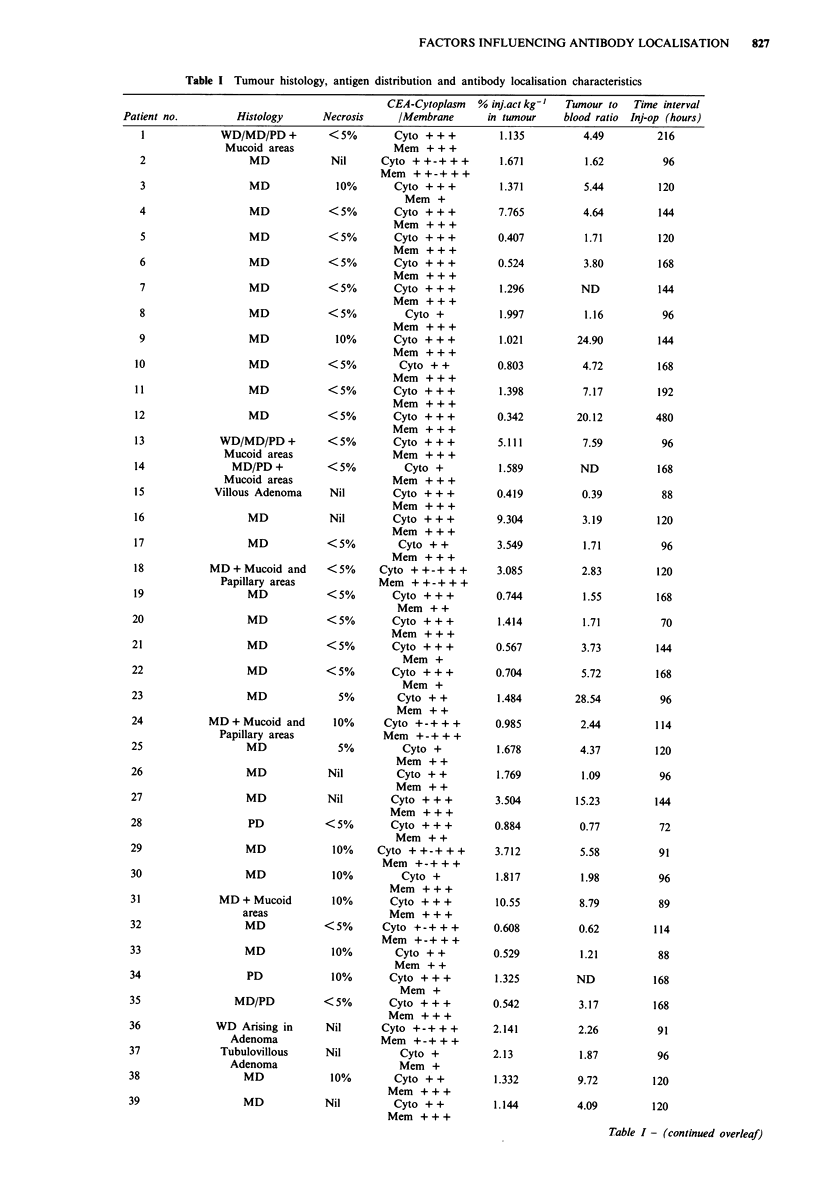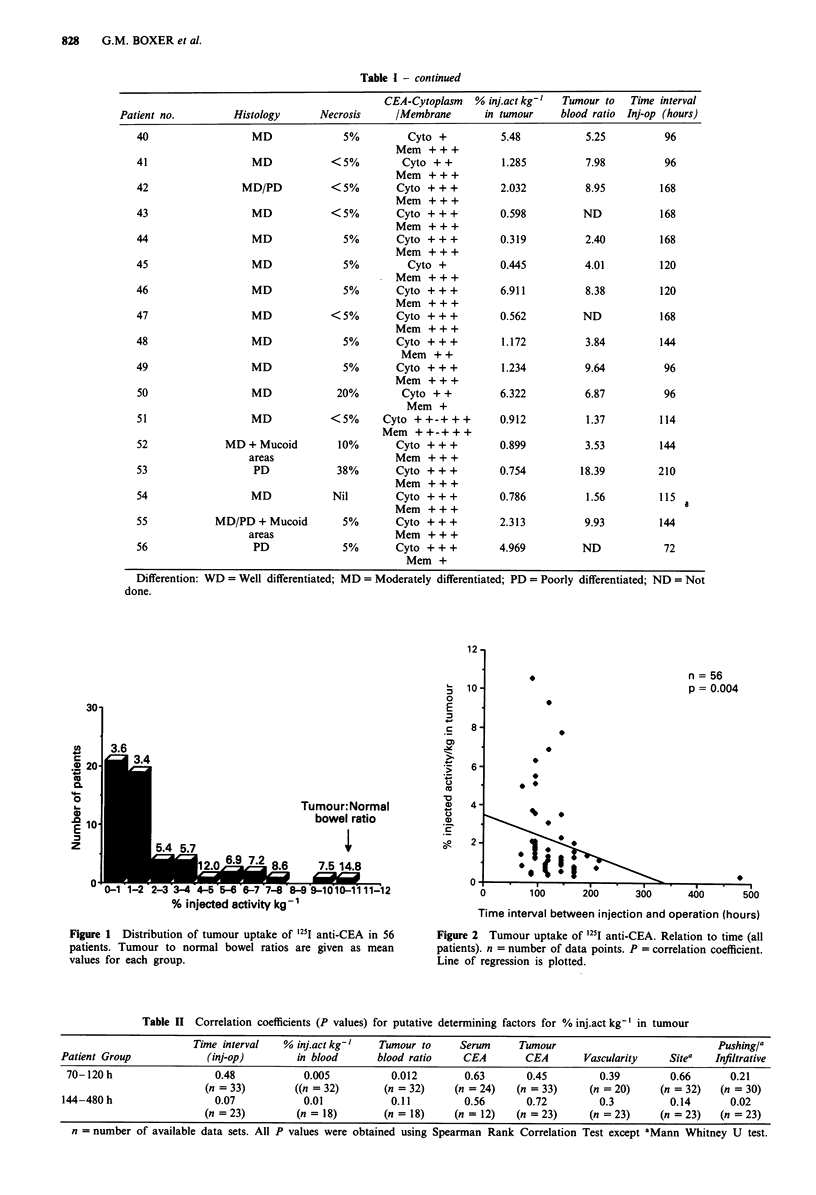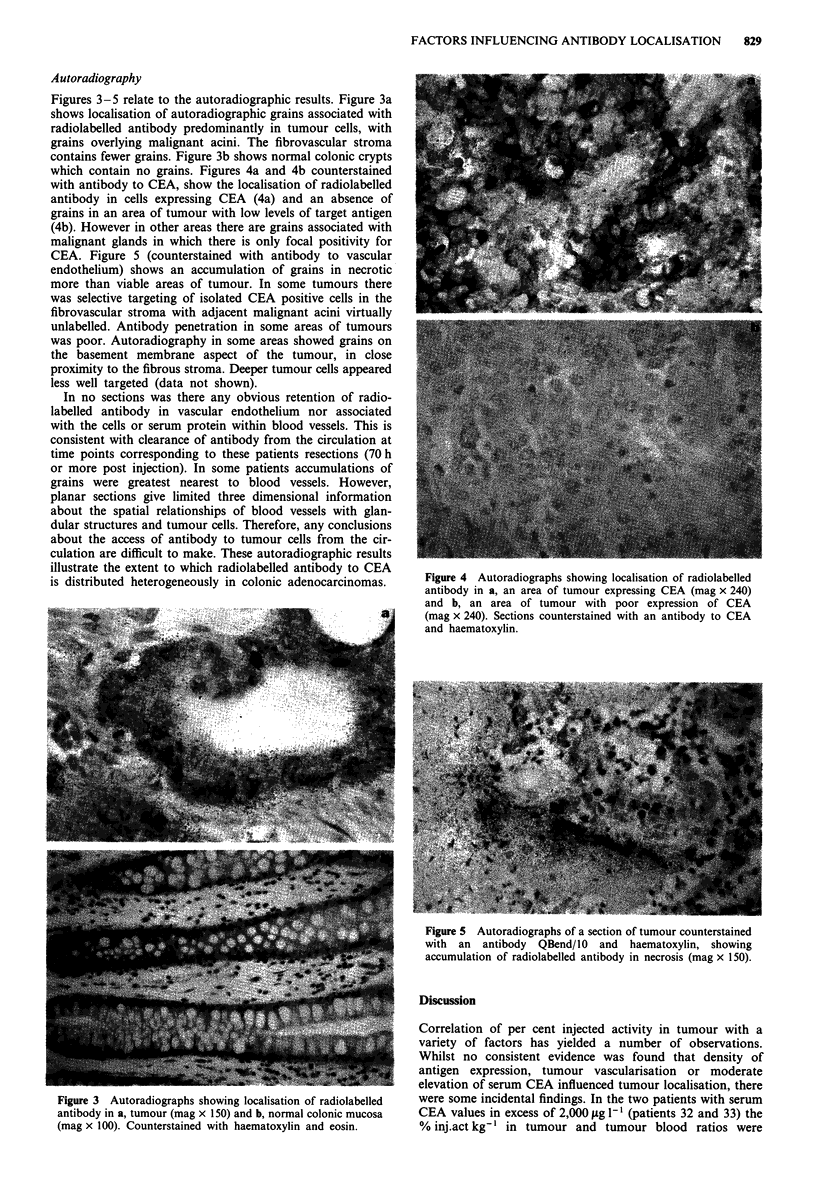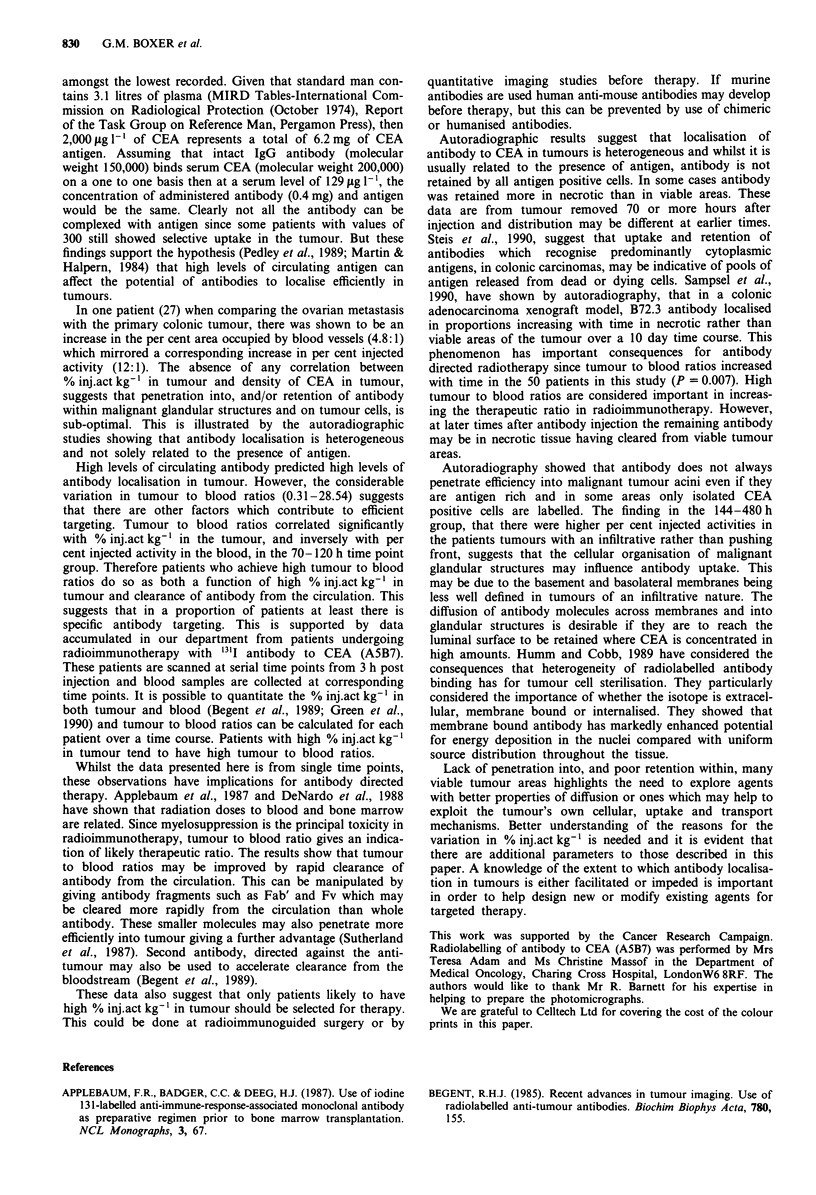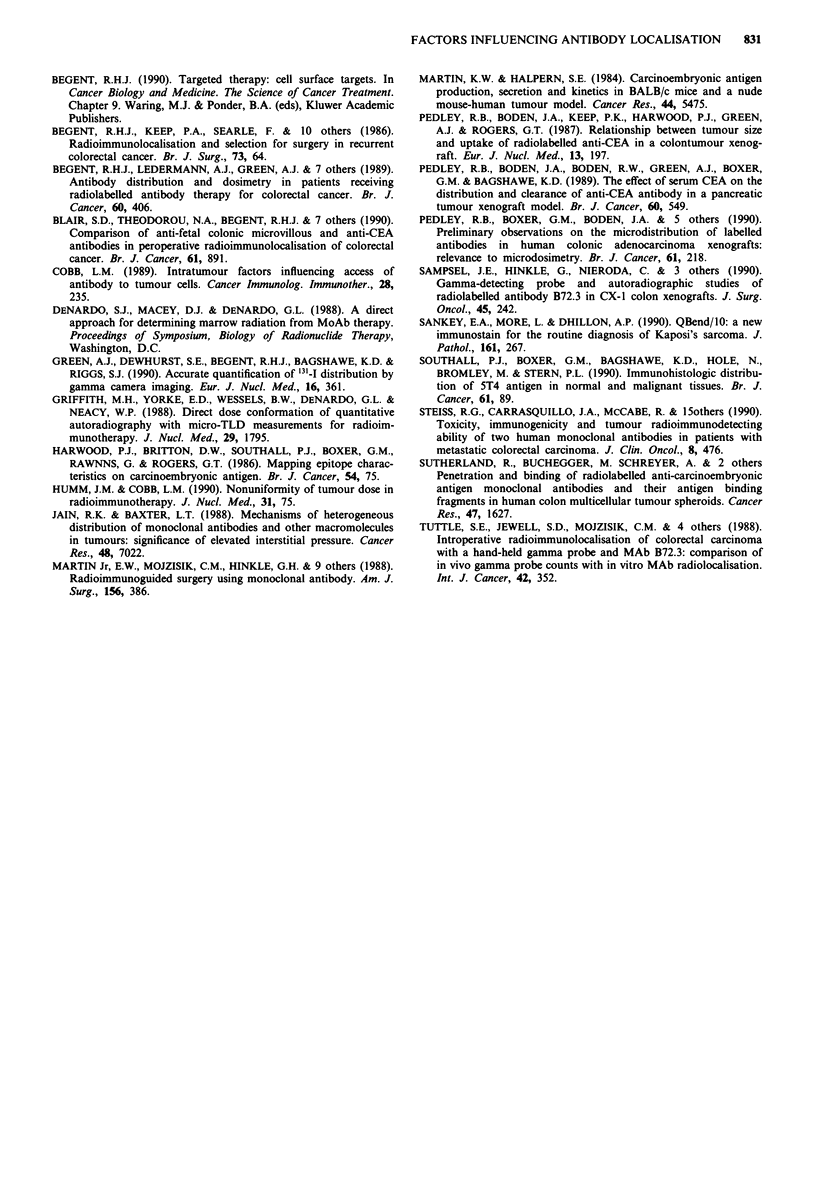# Factors influencing variability of localisation of antibodies to carcinoembryonic antigen (CEA) in patients with colorectal carcinoma--implications for radioimmunotherapy.

**DOI:** 10.1038/bjc.1992.176

**Published:** 1992-06

**Authors:** G. M. Boxer, R. H. Begent, A. M. Kelly, P. J. Southall, S. B. Blair, N. A. Theodorou, P. M. Dawson, J. A. Ledermann

**Affiliations:** University Department of Clinical Oncology, Royal Free Hospital School of Medicine, London, UK.

## Abstract

**Images:**


Br. J. Cancer (1992), 65, 825-831 © Macmillan Press Ltd., 1992

### Factors influencing variability of localisation of antibodies to carcinoembryonic antigen (CEA) in patients with colorectal carcinoma – implications for radioimmunotherapy

G.M. Boxer[1], R.H.J. Begent[1], A.M.B. Kelly[1], P.J. Southall[1], S.B. Blair[2], N.A. Theodorou[2], P.M. Dawson[2] & J.A. Ledermann[3]

[1]*Cancer Research Campaign Clinical Research Laboratories, University Department of Clinical Oncology, Royal Free Hospital School of Medicine, London NW3 2PF;* [2]*Department of Surgery, Charing Cross Hospital, London W6 8RF;* [3]*Department of Oncology, University College and Middlesex School of Medicine, London W1P 8BT, UK.*

**Summary** Tumour localisation of anti-tumour antibodies varies greatly between patients. Factors which may be responsible for this have been investigated in 56 patients with colorectal carcinoma with a view to improving radioimmunotherapy. Thirty-seven to seventy-four MBq of $^{125}$-I labelled mouse monoclonal antibody to CEA, was given intravenously and tumour resected 70–480 h later. Percentage injected activity $kg^{-1}$ (% inj.act $kg^{-1}$) in tumour, was inversely correlated with the time interval between injection and operation ($P = 0.004$). To assess the influence of other parameters on localisation, patients were divided into two time groups according to time interval between injection and operation, 70–120 h ($n = 33$) and 144–480 h ($n = 23$). In neither group was there a significant correlation of % inj.act $kg^{-1}$ with time. The % inj.act $kg^{-1}$ in tumour showed a significant correlation with that in the blood for both groups ($P = 0.005$ and $P = 0.01$). There was no significant correlation for either time group between % inj.act $kg^{-1}$ in tumour and serum CEA values, the per cent of tumour cells positive for CEA and vascularity. Tumour to blood ratios varied considerably (range 0.3–28.5:1) suggesting that factors other than time and persistence of activity in the blood contribute to efficient targeting. Tumour to blood ratios were inversely correlated with % inj.act $kg^{-1}$ in blood for the 70–120 h group ($P = 0.007$), and were positively correlated with % inj.act $kg^{-1}$ in tumour ($P = 0.012$).

Autoradiography showed that antibody localised predominantly on tumour cells but was distributed heterogeneously, was not solely related to the expression of antigen and in some cases accumulated in necrotic more than viable areas of tumour. Penetration of antibody into malignant acinar structures was poor and CEA-positive cells closer to the blood supply were targeted to a greater extent than distant cells. Preoperative administration of radiolabelled antibody to CEA may be helpful in selecting patients with favourable localisation for radioimmunotherapy.

Localisation of intravenously administered anti-tumour antibodies in tumours is used clinically in immunoscintigraphy (IS), radioimmunoguided surgery (RIGS) and anitbody targeted therapy of cancer (Begent *et al.*, 1985; Martin *et al.*, 1988; Begent, 1990). There is great variability in uptake of antibody in individual tumours in patients and the factors influencing this are poorly understood. Distribution of injected antibodies within tumours is not uniform (Griffith *et al.*, 1988; Pedley *et al.*, 1990). The causes of heterogeneity of intratumour distribution of antibody have been addressed by Cobb, 1989 and Jain and Baxter, 1988 and the consequences of nonuniformity of antibody binding on tumour dose in radioimmunotherapy are discussed by Humm and Cobb, 1990. Most autoradiographic studies of the distribution of antibody at the microscopic level have been confined to human tumour xenograft model systems in nude mice (Pedley *et al.*, 1990; Sampsel *et al.*, 1990). In patients, tumour and normal tissues from resected specimens collected following radioimmunoguided surgery with $^{125}$I-labelled antibody (Blair *et al.*, 1990), allows the microdistribution of antibody to be investigated. A number of factors may putatively influence antibody localisation, including antigen heterogeneity, tumour vascularisation and rate of clearance of antibody from the circulation.

This paper attempts to clarify how factors affecting biodistribution of antibody may be important in modifying existing agents or designing new ones for antibody targeted therapy, IS and RIGS. The preoperative administration of radiolabelled antibody for use with RIGS (Blair *et al.*, 1990; Tuttle *et al.*, 1988) provides an opportunity to obtain tissue samples from patients receiving antibody which are suitable for studying the relevant parameters. Autoradiography, combined with immunohistochemical demonstration of CEA and vascular endothelium, shows the relationship of localised antibody to the target antigen and tumour vasculature. Information about the extravasation, penetration, passage and retention of antibody within tumours and factors affecting localisation is essential if new or modified agents are to be designed for efficient antibody directed therapy of cancer.

### Patients and methods

Fifty-six patients with colorectal carcinoma gave informed consent to enter the trial. Antibody uptake in thyroid was blocked by administration of potassium iodide and potassium perchlorate. Patients were tested for skin allergy as previously described (Begent *et al.*, 1986). 0.4 mg of IgG1 mouse monoclonal antibody (A5B7) to CEA (Harwood *et al.*, 1986) was radiolabelled with of $^{125}$I, by the Chloramine T method over ice, to a specific activity of 93–185 MBq $mg^{-1}$ and injected intravenously. Patients underwent surgery 70–480 h (mean 135) later.

### *Gamma counting of radioactivity*

Resected specimens were collected and representative samples from the tumour, normal mucosa, and lymph nodes were taken. Where present any sites of metastatic tumour were dissected out. Blood samples were taken on the day of operation. Tissue and blood samples were weighed, dissolved in 2 ml of 7 M potassium hydroxide and counted for gamma radioactivity in a Compugamma (LKB). The percentage injected activity per kilogram of tissue was calculated (Pedley *et al.*, 1987). Adjacent pieces of tissue were fixed in 10% formalin and processed for routine histology.

Correspondence: G.M. Boxer, Department of Clinical Oncology, Royal Free Hospital School of Medicine, London NW3 2PF, UK.
Received 4 October 1991; and in revised form 31 January 1992.

*Immunohistochemistry for CEA antigen and tumour vessels*

Four micron sections of formalin-fixed paraffin processed tumour tissue were incubated with two mouse monoclonal antibodies to different epitopes on CEA, F3E3 and A5B7 (Harwood *et al.*, 1986) in an avidin biotin peroxidase technique (Southall *et al.*, 1990). As a measure of antigen density in the tumour the number of CEA positive cells were counted in 50 randomly chosen fields (× 200) and expressed as a percentage of total cells (per cent CEA + ve cells). In addition to tumour cells colorectal adenocarcinomas are partly composed of fibrovascular stroma which does not produce CEA. To account for this, in the same fields, the area of tumour (T) *vs* stroma (S) was calculated using an image analyser (Joyce-Loebl) by drawing around areas of tumour cells and expressed as a percentage (T/(T + S) × 100). A measure of the percentage of tumour containing CEA was calculated from (per cent CEA + ve cells) × (T/(T + S) × 100). The differentiation, site, amount of necrosis, and the pushing or infiltrative nature of the tumour were recorded. Necrotic areas were measured using the image analyser and expressed as a per cent of the tumour area. Necrotic material confined within complete, viable glands was not included. Antigen distribution within tumours, demonstrated by immunohistochemistry, was assessed in terms of binding to membrane or cytoplasm of cells and intensity of reaction was scored on an arbitrary scale of + to + + +.

Tumour blood vessels were demonstrated immunohistochemically as described above using an antibody to vascular endothelium, QBend/10 (Sankey *et al.*, 1990). Twenty-five fields (× 200) from areas of viable tumour were randomly selected in each patient. The percentage of the field occupied by blood vessels was calculated, using the image analyser to draw around the internal circumference of each vessel.

*Autoradiography*

Autoradiographic studies were performed in 12 cases in which sufficient radioactive counts were obtained in tumour (in excess of 100,000 counts per minute gram$^{-1}$). The technique was as previously described (Pedley *et al.*, 1990). Before covering with autoradiographic film, serial sections of tumour and normal tissues were immunohistochemically stained with an antibody to CEA and with QBend/10 as previously described in the manuscript. Control sections, incubated with biotinylated anti-mouse antibody and avidin biotin peroxidase alone, were always negative. Sections were counterstained after development of the autoradiographs with haematoxylin only.

*Statistical analysis*

Correlations of the per cent injected activity in tumour and tumour to blood ratios, with blood levels of radioactivity, serum CEA, tumour CEA, vascularity and the time interval between injection and operation were investigated. Correlations of parameters with numerical values were performed using the non parametric Spearman Rank Correlation Test. Correlations of per cent injected activity in tumour with site and the pushing or infiltrative nature of the tumour were performed using the Mann-Whitney U test.

### Results

*Tumour characteristics and antibody localisation*

The histological and immunohistochemical (distribution of CEA) characteristics of the tumours, the % inj.act kg$^{-1}$ in tumour, tumour to blood ratios and time interval between injection and operation are shown in Table I. The wide range of % inj.act kg$^{-1}$ in tumour (0.342–10.55) is illustrated. Most of the tumours were moderately differentiated. The amount of differentiation did not influence tumour uptake of radiolabelled antibody. In most patients, immunohistochemistry showed antigen in tumour to be both cytoplasmic and membrane associated with similar intensity of reaction. In those few patients whose tumours showed a differential intensity in reaction between cytoplasm and membrane there seemed to be no association with % inj.act kg$^{-1}$ in tumour or tumour to blood ratios. In 29/56 necrotic areas constituted less than 5% of total area and in 12/56 necrotic areas constituted 10% or more. These tumours were not associated with higher or lower per cent injected activities.

Figure 1 shows the distribution of tumour uptake (inj.act kg$^{-1}$) of $^{125}$I anti-CEA in the 56 patients. Whilst 40 of the 56 patients had % inj.act kg$^{-1}$ of less than 2% they achieved mean tumour to normal bowel ratios of 3.5. Although patients with more than 2% injected activities in tumour were less frequent they were associated with higher tumour to normal bowel ratios.

*Factors affecting % inj.act kg$^{-1}$ in tumour*

Table II shows the correlations of % inj.act kg$^{-1}$ in tumour with eight factors. The % inj.act kg$^{-1}$ in tumour was positively correlated with % inj.act kg$^{-1}$ in blood and, for the early time group, with tumour to blood ratio. The other factors had no significant effect.

Figure 2 shows a significant inverse relationship between % inj.act kg$^{-1}$ in tumour and the time interval between injection and operation in the 56 patients ($P = 0.004$). Becuase of this relationship of tumour uptake with time in the whole patient group, in order to investigate the influence of other parameters, the patients were divided into two subgroups according to the time interval between injection and operation. These were 70–120 h ($n = 33$) and 144–480 h ($n = 23$). In neither group was there a significant dependence upon time ($P = 0.48$ and $P = 0.07$).

There was a significant correlation between % inj.act kg$^{-1}$ in tumour and % inj.act kg$^{-1}$ in blood in the 70–120 h ($P = 0.005$) and the 144–480 h ($P = 0.01$) groups.

*Tumour to blood ratio*

There was a significant inverse relationship between % inj.act kg$^{-1}$ in blood and the tumour to blood ratio ($P = 0.007$) in the 70–120 h group. In the 144–480 h group the relationship did not achieve significance ($P = 0.08$). There was also a significant correlation between % inj.act kg$^{-1}$ in tumour and tumour to blood ratios ($P = 0.012$) for the 70–120 h group. In the 144–480 h group the correlation was not significant ($P = 0.11$).

*Other factors*

The relationship between % inj.act kg$^{-1}$ in tumour and serum CEA levels was not significant in either the early ($P = 0.63$) or late ($P = 0.56$) time point group. However, two patients with values in excess of 2,000 μg l$^{-1}$ showed low % inj.act kg$^{-1}$ in tumour (0.608 and 0.,529) and low tumour to blood ratios (0.6 and 1.2). There was no relationship between % inj.act kg$^{-1}$ in tumour and density of CEA in viable tumour in either the early ($P = 0.44$) or late ($P = 0.71$) time point group. There was no significant relationship between % inj.act kg$^{-1}$ in tumour and vascularity in viable tumour in either the early ($P = 0.39$) or late ($P = 0.28$) time point group. However, in one patient, increased localisation in an ovarian metastasis compared with the primary colonic tumour (12:1) was associated with an increase in vascularity (4.8:1), in the ovarian *vs* the colonic tumour. The density of CEA, demonstrated immunohistochemically in the tumour at each site, appeared similar. There was no association between the % inj.act kg$^{-1}$ in tumour and the site (colon or rectum), for the early ($P = 0.66$) or late ($P = 0.14$) time point groups. In the 144–480 h group the per cent injected activities were higher in the patients tumours with an infiltrative rather than pushing front ($P = 0.02$).

**Table I** Tumour histology, antigen distribution and antibody localisation characteristics

| *Patient no.* | *Histology* | *Necrosis* | *CEA-Cytoplasm /Membrane* | *% inj.act kg$^{-1}$ in tumour* | *Tumour to blood ratio* | *Time interval Inj-op (hours)* |
|---|---|---|---|---|---|---|
| 1 | WD/MD/PD + Mucoid areas | <5% | Cyto +++ Mem +++ | 1.135 | 4.49 | 216 |
| 2 | MD | Nil | Cyto ++-+++ Mem ++-+++ | 1.671 | 1.62 | 96 |
| 3 | MD | 10% | Cyto +++ Mem + | 1.371 | 5.44 | 120 |
| 4 | MD | <5% | Cyto +++ Mem +++ | 7.765 | 4.64 | 144 |
| 5 | MD | <5% | Cyto +++ Mem +++ | 0.407 | 1.71 | 120 |
| 6 | MD | <5% | Cyto +++ Mem +++ | 0.524 | 3.80 | 168 |
| 7 | MD | <5% | Cyto +++ Mem +++ | 1.296 | ND | 144 |
| 8 | MD | <5% | Cyto + Mem +++ | 1.997 | 1.16 | 96 |
| 9 | MD | 10% | Cyto +++ Mem +++ | 1.021 | 24.90 | 144 |
| 10 | MD | <5% | Cyto ++ Mem +++ | 0.803 | 4.72 | 168 |
| 11 | MD | <5% | Cyto +++ Mem +++ | 1.398 | 7.17 | 192 |
| 12 | MD | <5% | Cyto +++ Mem +++ | 0.342 | 20.12 | 480 |
| 13 | WD/MD/PD + Mucoid areas | <5% | Cyto +++ Mem +++ | 5.111 | 7.59 | 96 |
| 14 | MD/PD + Mucoid areas | <5% | Cyto + Mem +++ | 1.589 | ND | 168 |
| 15 | Villous Adenoma | Nil | Cyto +++ Mem +++ | 0.419 | 0.39 | 88 |
| 16 | MD | Nil | Cyto +++ Mem +++ | 9.304 | 3.19 | 120 |
| 17 | MD | <5% | Cyto ++ Mem +++ | 3.549 | 1.71 | 96 |
| 18 | MD + Mucoid and Papillary areas | <5% | Cyto ++-+++ Mem ++-+++ | 3.085 | 2.83 | 120 |
| 19 | MD | <5% | Cyto +++ Mem ++ | 0.744 | 1.55 | 168 |
| 20 | MD | <5% | Cyto +++ Mem +++ | 1.414 | 1.71 | 70 |
| 21 | MD | <5% | Cyto +++ Mem + | 0.567 | 3.73 | 144 |
| 22 | MD | <5% | Cyto +++ Mem + | 0.704 | 5.72 | 168 |
| 23 | MD | 5% | Cyto ++ Mem ++ | 1.484 | 28.54 | 96 |
| 24 | MD + Mucoid and Papillary areas | 10% | Cyto +-+++ Mem +-+++ | 0.985 | 2.44 | 114 |
| 25 | MD | 5% | Cyto + Mem ++ | 1.678 | 4.37 | 120 |
| 26 | MD | Nil | Cyto ++ Mem ++ | 1.769 | 1.09 | 96 |
| 27 | MD | Nil | Cyto +++ Mem +++ | 3.504 | 15.23 | 144 |
| 28 | PD | <5% | Cyto +++ Mem ++ | 0.884 | 0.77 | 72 |
| 29 | MD | 10% | Cyto ++-+++ Mem +-+++ | 3.712 | 5.58 | 91 |
| 30 | MD | 10% | Cyto + Mem +++ | 1.817 | 1.98 | 96 |
| 31 | MD + Mucoid areas | 10% | Cyto +++ Mem +++ | 10.55 | 8.79 | 89 |
| 32 | MD | <5% | Cyto +-+++ Mem +-+++ | 0.608 | 0.62 | 114 |
| 33 | MD | 10% | Cyto ++ Mem ++ | 0.529 | 1.21 | 88 |
| 34 | PD | 10% | Cyto +++ Mem + | 1.325 | ND | 168 |
| 35 | MD/PD | <5% | Cyto +++ Mem +++ | 0.542 | 3.17 | 168 |
| 36 | WD Arising in Adenoma | Nil | Cyto +-+++ Mem +-+++ | 2.141 | 2.26 | 91 |
| 37 | Tubulovillous Adenoma | Nil | Cyto + Mem + | 2.13 | 1.87 | 96 |
| 38 | MD | 10% | Cyto ++ Mem +++ | 1.332 | 9.72 | 120 |
| 39 | MD | Nil | Cyto ++ Mem +++ | 1.144 | 4.09 | 120 |

*Table I – (continued overleaf)*

**Table I** – *continued*

| Patient no. | Histology | Necrosis | CEA-Cytoplasm /Membrane | % inj.act $kg^{-1}$ in tumour | Tumour to blood ratio | Time interval Inj-op (hours) |
|---|---|---|---|---|---|---|
| 40 | MD | 5% | Cyto + Mem + + + | 5.48 | 5.25 | 96 |
| 41 | MD | <5% | Cyto + + Mem + + + | 1.285 | 7.98 | 96 |
| 42 | MD/PD | <5% | Cyto + + + Mem + + + | 2.032 | 8.95 | 168 |
| 43 | MD | <5% | Cyto + + + Mem + + + | 0.598 | ND | 168 |
| 44 | MD | 5% | Cyto + + + Mem + + + | 0.319 | 2.40 | 168 |
| 45 | MD | 5% | Cyto + Mem + + + | 0.445 | 4.01 | 120 |
| 46 | MD | 5% | Cyto + + + Mem + + + | 6.911 | 8.38 | 120 |
| 47 | MD | <5% | Cyto + + + Mem + + + | 0.562 | ND | 168 |
| 48 | MD | 5% | Cyto + + + Mem + + | 1.172 | 3.84 | 144 |
| 49 | MD | 5% | Cyto + + + Mem + + + | 1.234 | 9.64 | 96 |
| 50 | MD | 20% | Cyto + + Mem + | 6.322 | 6.87 | 96 |
| 51 | MD | <5% | Cyto + +-+ + + Mem + +-+ + + | 0.912 | 1.37 | 114 |
| 52 | MD + Mucoid areas | 10% | Cyto + + + Mem + + + | 0.899 | 3.53 | 144 |
| 53 | PD | 38% | Cyto + + + Mem + + + | 0.754 | 18.39 | 210 |
| 54 | MD | Nil | Cyto + + + Mem + + + | 0.786 | 1.56 | 115 |
| 55 | MD/PD + Mucoid areas | 5% | Cyto + + + Mem + + + | 2.313 | 9.93 | 144 |
| 56 | PD | 5% | Cyto + + + Mem + | 4.969 | ND | 72 |

Differention: WD = Well differentiated; MD = Moderately differentiated; PD = Poorly differentiated; ND = Not done.

**Figure 1** Distribution of tumour uptake of $^{125}I$ anti-CEA in 56 patients. Tumour to normal bowel ratios are given as mean values for each group.

**Figure 2** Tumour uptake of $^{125}I$ anti-CEA. Relation to time (all patients). $n$ = number of data points. $P$ = correlation coefficient. Line of regression is plotted.

**Table II** Correlation coefficients ($P$ values) for putative determining factors for % inj.act $kg^{-1}$ in tumour

| Patient Group | Time interval (inj-op) | % inj.act $kg^{-1}$ in blood | Tumour to blood ratio | Serum CEA | Tumour CEA | Vascularity | Site[a] | Pushing/[a] Infiltrative |
|---|---|---|---|---|---|---|---|---|
| 70–120 h | 0.48 ($n$ = 33) | 0.005 (($n$ = 32) | 0.012 ($n$ = 32) | 0.63 ($n$ = 24) | 0.45 ($n$ = 33) | 0.39 ($n$ = 20) | 0.66 ($n$ = 32) | 0.21 ($n$ = 30) |
| 144–480 h | 0.07 ($n$ = 23) | 0.01 ($n$ = 18) | 0.11 ($n$ = 18) | 0.56 ($n$ = 12) | 0.72 ($n$ = 23) | 0.3 ($n$ = 23) | 0.14 ($n$ = 23) | 0.02 ($n$ = 23) |

$n$ = number of available data sets. All $P$ values were obtained using Spearman Rank Correlation Test except [a]Mann Whitney U test.

*Autoradiography*

Figures 3–5 relate to the autoradiographic results. Figure 3a shows localisation of autoradiographic grains associated with radiolabelled antibody predominantly in tumour cells, with grains overlying malignant acini. The fibrovascular stroma contains fewer grains. Figure 3b shows normal colonic crypts which contain no grains. Figures 4a and 4b counterstained with antibody to CEA, show the localisation of radiolabelled antibody in cells expressing CEA (4a) and an absence of grains in an area of tumour with low levels of target antigen (4b). However in other areas there are grains associated with malignant glands in which there is only focal positivity for CEA. Figure 5 (counterstained with antibody to vascular endothelium) shows an accumulation of grains in necrotic more than viable areas of tumour. In some tumours there was selective targeting of isolated CEA positive cells in the fibrovascular stroma with adjacent malignant acini virtually unlabelled. Antibody penetration in some areas of tumours was poor. Autoradiography in some areas showed grains on the basement membrane aspect of the tumour, in close proximity to the fibrous stroma. Deeper tumour cells appeared less well targeted (data not shown).

In no sections was there any obvious retention of radiolabelled antibody in vascular endothelium nor associated with the cells or serum protein within blood vessels. This is consistent with clearance of antibody from the circulation at time points corresponding to these patients resections (70 h or more post injection). In some patients accumulations of grains were greatest nearest to blood vessels. However, planar sections give limited three dimensional information about the spatial relationships of blood vessels with glandular structures and tumour cells. Therefore, any conclusions about the access of antibody to tumour cells from the circulation are difficult to make. These autoradiographic results illustrate the extent to which radiolabelled antibody to CEA is distributed heterogeneously in colonic adenocarcinomas.

**Figure 3** Autoradiographs showing localisation of radiolabelled antibody in **a**, tumour (mag × 150) and **b**, normal colonic mucosa (mag × 100). Counterstained with haematoxylin and eosin.

**Figure 4** Autoradiographs showing localisation of radiolabelled antibody in **a**, an area of tumour expressing CEA (mag × 240) and **b**, an area of tumour with poor expression of CEA (mag × 240). Sections counterstained with an antibody to CEA and haematoxylin.

**Figure 5** Autoradiographs of a section of tumour counterstained with an antibody QBend/10 and haematoxylin, showing accumulation of radiolabelled antibody in necrosis (mag × 150).

### Discussion

Correlation of per cent injected activity in tumour with a variety of factors has yielded a number of observations. Whilst no consistent evidence was found that density of antigen expression, tumour vascularisation or moderate elevation of serum CEA influenced tumour localisation, there were some incidental findings. In the two patients with serum CEA values in excess of 2,000 $\mu g\, l^{-1}$ (patients 32 and 33) the % inj.act $kg^{-1}$ in tumour and tumour blood ratios were amongst the lowest recorded. Given that standard man contains 3.1 litres of plasma (MIRD Tables-International Commission on Radiological Protection (October 1974), Report of the Task Group on Reference Man, Pergamon Press), then 2,000 $\mu g l^{-1}$ of CEA represents a total of 6.2 mg of CEA antigen. Assuming that intact IgG antibody (molecular weight 150,000) binds serum CEA (molecular weight 200,000) on a one to one basis then at a serum level of 129 $\mu g l^{-1}$, the concentration of administered antibody (0.4 mg) and antigen would be the same. Clearly not all the antibody can be complexed with antigen since some patients with values of 300 still showed selective uptake in the tumour. But these findings support the hypothesis (Pedley *et al.*, 1989; Martin & Halpern, 1984) that high levels of circulating antigen can affect the potential of antibodies to localise efficiently in tumours.

In one patient (27) when comparing the ovarian metastasis with the primary colonic tumour, there was shown to be an increase in the per cent area occupied by blood vessels (4.8:1) which mirrored a corresponding increase in per cent injected activity (12:1). The absence of any correlation between % inj.act $kg^{-1}$ in tumour and density of CEA in tumour, suggests that penetration into, and/or retention of antibody within malignant glandular structures and on tumour cells, is sub-optimal. This is illustrated by the autoradiographic studies showing that antibody localisation is heterogeneous and not solely related to the presence of antigen.

High levels of circulating antibody predicted high levels of antibody localisation in tumour. However, the considerable variation in tumour to blood ratios (0.31–28.54) suggests that there are other factors which contribute to efficient targeting. Tumour to blood ratios correlated significantly with % inj.act $kg^{-1}$ in the tumour, and inversely with per cent injected activity in the blood, in the 70–120 h time point group. Therefore patients who achieve high tumour to blood ratios do so as both a function of high % inj.act $kg^{-1}$ in tumour and clearance of antibody from the circulation. This suggests that in a proportion of patients at least there is specific antibody targeting. This is supported by data accumulated in our department from patients undergoing radioimmunotherapy with $^{131}I$ antibody to CEA (A5B7). These patients are scanned at serial time points from 3 h post injection and blood samples are collected at corresponding time points. It is possible to quantitate the % inj.act $kg^{-1}$ in both tumour and blood (Begent *et al.*, 1989; Green *et al.*, 1990) and tumour to blood ratios can be calculated for each patient over a time course. Patients with high % inj.act $kg^{-1}$ in tumour tend to have high tumour to blood ratios.

Whilst the data presented here is from single time points, these observations have implications for antibody directed therapy. Applebaum *et al.*, 1987 and DeNardo *et al.*, 1988 have shown that radiation doses to blood and bone marrow are related. Since myelosuppression is the principal toxicity in radioimmunotherapy, tumour to blood ratio gives an indication of likely therapeutic ratio. The results show that tumour to blood ratios may be improved by rapid clearance of antibody from the circulation. This can be manipulated by giving antibody fragments such as Fab′ and Fv which may be cleared more rapidly from the circulation than whole antibody. These smaller molecules may also penetrate more efficiently into tumour giving a further advantage (Sutherland *et al.*, 1987). Second antibody, directed against the anti-tumour may also be used to accelerate clearance from the bloodstream (Begent *et al.*, 1989).

These data also suggest that only patients likely to have high % inj.act $kg^{-1}$ in tumour should be selected for therapy. This could be done at radioimmunoguided surgery or by quantitative imaging studies before therapy. If murine antibodies are used human anti-mouse antibodies may develop before therapy, but this can be prevented by use of chimeric or humanised antibodies.

Autoradiographic results suggest that localisation of antibody to CEA in tumours is heterogeneous and whilst it is usually related to the presence of antigen, antibody is not retained by all antigen positive cells. In some cases antibody was retained more in necrotic than in viable areas. These data are from tumour removed 70 or more hours after injection and distribution may be different at earlier times. Steis *et al.*, 1990, suggest that uptake and retention of antibodies which recognise predominantly cytoplasmic antigens, in colonic carcinomas, may be indicative of pools of antigen released from dead or dying cells. Sampsel *et al.*, 1990, have shown by autoradiography, that in a colonic adenocarcinoma xenograft model, B72.3 antibody localised in proportions increasing with time in necrotic rather than viable areas of the tumour over a 10 day time course. This phenomenon has important consequences for antibody directed radiotherapy since tumour to blood ratios increased with time in the 50 patients in this study ($P = 0.007$). High tumour to blood ratios are considered important in increasing the therapeutic ratio in radioimmunotherapy. However, at later times after antibody injection the remaining antibody may be in necrotic tissue having cleared from viable tumour areas.

Autoradiography showed that antibody does not always penetrate efficiency into malignant tumour acini even if they are antigen rich and in some areas only isolated CEA positive cells are labelled. The finding in the 144–480 h group, that there were higher per cent injected activities in the patients tumours with an infiltrative rather than pushing front, suggests that the cellular organisation of malignant glandular structures may influence antibody uptake. This may be due to the basement and basolateral membranes being less well defined in tumours of an infiltrative nature. The diffusion of antibody molecules across membranes and into glandular structures is desirable if they are to reach the luminal surface to be retained where CEA is concentrated in high amounts. Humm and Cobb, 1989 have considered the consequences that heterogeneity of radiolabelled antibody binding has for tumour cell sterilisation. They particularly considered the importance of whether the isotope is extracellular, membrane bound or internalised. They showed that membrane bound antibody has markedly enhanced potential for energy deposition in the nuclei compared with uniform source distribution throughout the tissue.

Lack of penetration into, and poor retention within, many viable tumour areas highlights the need to explore agents with better properties of diffusion or ones which may help to exploit the tumour's own cellular, uptake and transport mechanisms. Better understanding of the reasons for the variation in % inj.act $kg^{-1}$ is needed and it is evident that there are additional parameters to those described in this paper. A knowledge of the extent to which antibody localisation in tumours is either facilitated or impeded is important in order to help design new or modify existing agents for targeted therapy.

This work was supported by the Cancer Research Campaign. Radiolabelling of antibody to CEA (A5B7) was performed by Mrs Teresa Adam and Ms Christine Massof in the Department of Medical Oncology, Charing Cross Hospital, LondonW6 8RF. The authors would like to thank Mr R. Barnett for his expertise in helping to prepare the photomicrographs.

We are grateful to Celltech Ltd for covering the cost of the colour prints in this paper.

### References

APPLEBAUM, F.R., BADGER, C.C. & DEEG, H.J. (1987). Use of iodine 131-labelled anti-immune-response-associated monoclonal antibody as preparative regimen prior to bone marrow transplantation. *NCL Monographs*, **3**, 67.

BEGENT, R.H.J. (1985). Recent advances in tumour imaging. Use of radiolabelled anti-tumour antibodies. *Biochim Biophys Acta*, **780**, 155.

BEGENT, R.H.J. (1990). Targeted therapy: cell surface targets. In *Cancer Biology and Medicine. The Science of Cancer Treatment.* Chapter 9. Waring, M.J. & Ponder, B.A. (eds), Kluwer Academic Publishers.

BEGENT, R.H.J., KEEP, P.A., SEARLE, F. & 10 others (1986). Radioimmunolocalisation and selection for surgery in recurrent colorectal cancer. *Br. J. Surg.*, **73**, 64.

BEGENT, R.H.J., LEDERMANN, A.J., GREEN, A.J. & 7 others (1989). Antibody distribution and dosimetry in patients receiving radiolabelled antibody therapy for colorectal cancer. *Br. J. Cancer*, **60**, 406.

BLAIR, S.D., THEODOROU, N.A., BEGENT, R.H.J. & 7 others (1990). Comparison of anti-fetal colonic microvillous and anti-CEA antibodies in peroperative radioimmunolocalisation of colorectal cancer. *Br. J. Cancer*, **61**, 891.

COBB, L.M. (1989). Intratumour factors influencing access of antibody to tumour cells. *Cancer Immunolog. Immunother.*, **28**, 235.

DENARDO, S.J., MACEY, D.J. & DENARDO, G.L. (1988). A direct approach for determining marrow radiation from MoAb therapy. *Proceedings of Symposium, Biology of Radionuclide Therapy*, Washington, D.C.

GREEN, A.J., DEWHURST, S.E., BEGENT, R.H.J., BAGSHAWE, K.D. & RIGGS, S.J. (1990). Accurate quantification of $^{131}$-I distribution by gamma camera imaging. *Eur. J. Nucl. Med.*, **16**, 361.

GRIFFITH, M.H., YORKE, E.D., WESSELS, B.W., DENARDO, G.L. & NEACY, W.P. (1988). Direct dose conformation of quantitative autoradiography with micro-TLD measurements for radioimmunotherapy. *J. Nucl. Med.*, **29**, 1795.

HARWOOD, P.J., BRITTON, D.W., SOUTHALL, P.J., BOXER, G.M., RAWNNS, G. & ROGERS, G.T. (1986). Mapping epitope characteristics on carcinoembryonic antigen. *Br. J. Cancer*, **54**, 75.

HUMM, J.M. & COBB, L.M. (1990). Nonuniformity of tumour dose in radioimmunotherapy. *J. Nucl. Med.*, **31**, 75.

JAIN, R.K. & BAXTER, L.T. (1988). Mechanisms of heterogeneous distribution of monoclonal antibodies and other macromolecules in tumours: significance of elevated interstitial pressure. *Cancer Res.*, **48**, 7022.

MARTIN Jr, E.W., MOJZISIK, C.M., HINKLE, G.H. & 9 others (1988). Radioimmunoguided surgery using monoclonal antibody. *Am. J. Surg.*, **156**, 386.

MARTIN, K.W. & HALPERN, S.E. (1984). Carcinoembryonic antigen production, secretion and kinetics in BALB/c mice and a nude mouse-human tumour model. *Cancer Res.*, **44**, 5475.

PEDLEY, R.B., BODEN, J.A., KEEP, P.K., HARWOOD, P.J., GREEN, A.J. & ROGERS, G.T. (1987). Relationship between tumour size and uptake of radiolabelled anti-CEA in a colontumour xenograft. *Eur. J. Nucl. Med.*, **13**, 197.

PEDLEY, R.B., BODEN, J.A., BODEN, R.W., GREEN, A.J., BOXER, G.M. & BAGSHAWE, K.D. (1989). The effect of serum CEA on the distribution and clearance of anti-CEA antibody in a pancreatic tumour xenograft model. *Br. J. Cancer*, **60**, 549.

PEDLEY, R.B., BOXER, G.M., BODEN, J.A. & 5 others (1990). Preliminary observations on the microdistribution of labelled antibodies in human colonic adenocarcinoma xenografts: relevance to microdosimetry. *Br. J. Cancer*, **61**, 218.

SAMPSEL, J.E., HINKLE, G., NIERODA, C. & 3 others (1990). Gamma-detecting probe and autoradiographic studies of radiolabelled antibody B72.3 in CX-1 colon xenografts. *J. Surg. Oncol.*, **45**, 242.

SANKEY, E.A., MORE, L. & DHILLON, A.P. (1990). QBend/10: a new immunostain for the routine diagnosis of Kaposi's sarcoma. *J. Pathol.*, **161**, 267.

SOUTHALL, P.J., BOXER, G.M., BAGSHAWE, K.D., HOLE, N., BROMLEY, M. & STERN, P.L. (1990). Immunohistologic distribution of 5T4 antigen in normal and malignant tissues. *Br. J. Cancer*, **61**, 89.

STEISS, R.G., CARRASQUILLO, J.A., MCCABE, R. & 15others (1990). Toxicity, immunogenicity and tumour radioimmunodetecting ability of two human monoclonal antibodies in patients with metastatic colorectal carcinoma. *J. Clin. Oncol.*, **8**, 476.

SUTHERLAND, R., BUCHEGGER, M. SCHREYER, A. & 2 others Penetration and binding of radiolabelled anti-carcinoembryonic antigen monoclonal antibodies and their antigen binding fragments in human colon multicellular tumour spheroids. *Cancer Res.*, **47**, 1627.

TUTTLE, S.E., JEWELL, S.D., MOJZISIK, C.M. & 4 others (1988). Introperative radioimmunolocalisation of colorectal carcinoma with a hand-held gamma probe and MAb B72.3: comparison of in vivo gamma probe counts with in vitro MAb radiolocalisation. *Int. J. Cancer*, **42**, 352.